# Anisotropic engineered heart tissue made from laser-cut decellularized myocardium

**DOI:** 10.1038/srep32068

**Published:** 2016-08-30

**Authors:** Jonas Schwan, Andrea T. Kwaczala, Thomas J. Ryan, Oscar Bartulos, Yongming Ren, Lorenzo R. Sewanan, Aaron H. Morris, Daniel L. Jacoby, Yibing Qyang, Stuart G. Campbell

**Affiliations:** 1 Department of Biomedical Engineering, Yale University, New Haven, CT 06510, USA; 2 Department of Biomedical Engineering, University of Hartford, West Hartford, CT 06117, USA; 3 Yale Cardiovascular Research Center, Section of Cardiovascular Medicine, Department of Internal Medicine, Yale School of Medicine, New Haven, CT 06510, USA; 4 Yale Stem Cell Center, Yale University, New Haven, CT 06510, USA; 5 Vascular Biology and Therapeutics Program, Yale School of Medicine, New Haven, CT 06510, USA; 6 Department of Pathology, Yale University, New Haven, CT 06510, USA; 7 Department of Cellular and Molecular Physiology, Yale School of Medicine, New Haven, CT 06510, USA

## Abstract

We have developed an engineered heart tissue (EHT) system that uses laser-cut sheets of decellularized myocardium as scaffolds. This material enables formation of thin muscle strips whose biomechanical characteristics are easily measured and manipulated. To create EHTs, sections of porcine myocardium were laser-cut into ribbon-like shapes, decellularized, and mounted in specialized clips for seeding and culture. Scaffolds were first tested by seeding with neonatal rat ventricular myocytes. EHTs beat synchronously by day five and exhibited robust length-dependent activation by day 21. Fiber orientation within the scaffold affected peak twitch stress, demonstrating its ability to guide cells toward physiologic contractile anisotropy. Scaffold anisotropy also made it possible to probe cellular responses to stretch as a function of fiber angle. Stretch that was aligned with the fiber direction increased expression of brain natriuretic peptide, but off-axis stretches (causing fiber shear) did not. The method also produced robust EHTs from cardiomyocytes derived from human embryonic stem cells and induced pluripotent stem cells (hiPSC). hiPSC-EHTs achieved maximum peak stress of 6.5 mN/mm^2^ and twitch kinetics approaching reported values from adult human trabeculae. We conclude that laser-cut EHTs are a viable platform for novel mechanotransduction experiments and characterizing the biomechanical function of patient-derived cardiomyoctyes.

Abnormal growth and remodeling of the myocardium are central features in many heart disorders, including hypertensive heart disease, myocardial infarction, and inherited cardiomyopathies^1^. Such phenomena are challenging to study because of the many different factors that can perturb cardiac tissue homeostasis *in vivo*. These can include gene mutations, endocrine factors, and mechanical loads applied to the tissue^2^. Engineered heart tissues (EHTs) could be transformative *in vitro* tools for studying the remodeling process, because they allow the various contributing factors to be manipulated systematically in a controlled environment^3^,^4^,^5^,^6^,^7^.

In order to be most useful for cardiac remodeling studies, EHTs should have an extracellular matrix (ECM) with realistic molecular composition and three-dimensional microstructure. Cardiac cells sense their mechanical environment through structures that link the cytoskeleton to specific ECM components^8^,^9^. Therefore, an ECM composition that provides the proper set of binding partners for cells could be a critical factor in recapitulating *in vivo* mechanical signaling. Besides composition, the microstructural arrangement of ECM proteins is likely to be a key consideration. In healthy myocardium, cardiomyocytes are aligned to form a local prevailing fiber direction. The passive and active material properties of the myocardium depend strongly on whether they are measured parallel or perpendicular to these fibers. That is to say, the myocardium is highly anisotropic^10^. This suggests that mechanosensitive responses in cardiac tissue should also be studied in the context of carefully controlled material anisotropy.

Decellularized adult myocardium is a scaffold that provides realistic anisotropy and matrix composition. It is produced by treating tissue with chemicals that remove intracellular contents, leaving only the native extracellular matrix behind. Ott and co-workers^11^,^12^ used this pioneering approach to produce functioning cardiac tissue by seeding neonatal rat ventricular myocytes (NRVMs) as well as human induced pluripotent stem cell-derived cardiomyocytes (hiPSC-CM) into perfusion-decellularized hearts. Lu *et al.*^13^ later demonstrated the ability of decellularized mouse myocardium to promote differentiation and assembly of human cardiovascular progenitor cells into viable cardiac tissue. More recently, thick slices of decellularized myocardium have been immobilized on cover glass and seeded with NRVMs to produce tissue sheets with anisotropic action potential conduction^14^. These studies and others highlight the clear potential of decellularized myocardium to produce cardiac tissues with realistic anisotropy, but until now this technology has not been adapted for biomechanical experiments, which require the ability to both measure and manipulate mechanical loading of EHTs in precise ways.

Here, we report two key advances that have enabled us to more fully harness the potential of decellularized myocardium. After cutting thin cryosections of adult porcine myocardium, we have successfully used laser cutting to generate scaffolds with customizable, engineered shapes (Fig. 1a). We have also developed an innovative clipping system that mounts the ribbon-like scaffolds consistently and securely into a tissue culture ‘cassette’ (Fig. 1b). Scaffolds are then decellularized and reseeded with human or rat cardiac cells, ultimately yielding robust EHTs that are well suited for several biomechanical assays. The process of laser cutting allows us to precisely orient cardiac fibers relative to the macroscopic dimensions of the final construct. The clipping system enables precise application of novel mechanical perturbations (such as shear strain) and makes measurements of mechanical function rapid and reliable.

## Methods

All animal procedures were approved by the Yale University Institutional Animal Care and Use Committee. Development and use of human cell lines was performed under protocols approved by the Yale Institutional Review Board. All experiments were conducted in accordance with the relevant guidelines and established standards. Informed consent was obtained from all human subjects. Detailed descriptions of the decellularization procedure, calcium transient measurements, Real Time-quantitative PCR, primer sequences and creation of anisotropic electrospun gelatin scaffolds can be found in the Supplementary Methods.

### Tissue culture cassette

**Figure 1:**
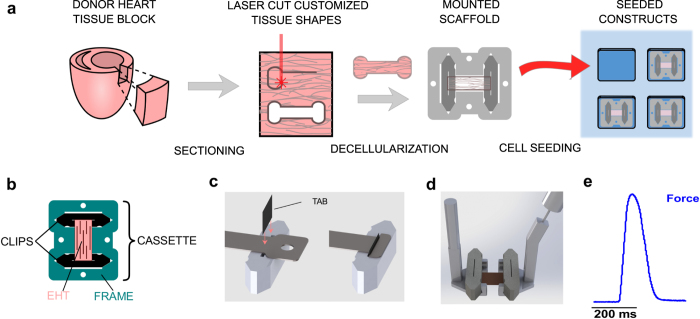
Steps in laser-cut scaffold generation. (**a**) Schematic diagram of the key steps in producing laser-cut EHTs. (**b**) Tissue culture cassette, which consists of PTFE clips (black) and frame (teal) assembly, used to culture EHTs (pink). (**c**) Schematic of the clamping method used to anchor the decellularized scaffold into the clip by means of a small plastic tab. (**d**) Computer rendering of the mechanical testing apparatus. An EHT (pink) attached to its culture clips is picked up by two claw-like extensions, one of which is attached to a force transducer (right side) that measures uniaxial force. (**e**) Representative force (blue) trace recorded from an EHT containing NRVMs under 0.5 Hz electrical stimulation at 37 °C bath temperature (scale bar, 200 milliseconds).

We designed an *in vitro* culturing system that protects the tissue during decellularization, allows the application of precise mechanical boundary conditions, facilitates repetitive, non-damaging transfer between culture and biomechanical testing apparatus, and allows for straightforward force measurements. The final tissue culture cassette design consists of two clips that mount into a frame as depicted in Fig. 1b. This ensures that the scaffold is held securely at a constant length throughout seeding and culture. Tissue culture cassettes were cut out of Polytetrafluoroethylene (PTFE), which allowed them to be biocompatible, autoclavable, and reusable.

### Generation of thin myocardial slices

Blocks of myocardium were dissected from the left ventricular free walls of young porcine hearts and flash frozen in powdered dry ice. Slices (150 μm-thick) were cut from each tissue block using a cryostat microtome. Blocks were oriented so that the cutting face was roughly parallel to the epicardial surface, ensuring that native cardiac fibers lay in the section plane. Sections were always taken from the mid-myocardium, since mechanical properties of the tissue at different wall depths are known to vary significantly^15^. Cut slices were stored at −80 °C for up to two months.

### Generation of customized shapes using laser cutting

**Figure 2:**
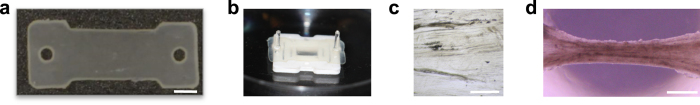
Images of intermediate protocol steps. (**a**) A laser-cut porcine scaffold prior to decellularization (scale bar, 2 mm). (**b**) Using the laser-cut holes within the scaffold, tissues are temporarily mounted onto removable posts placed into holes cut into either end of the culture frame (**c**) Native myocardial fiber orientation (running left to right) can be seen by light micrograph after decellularization (scale bar, 250 μm). (**d**) After seeding with NRVMs and 16 days in culture, EHTs form hourglass shapes and exhibit spontaneous beating (scale bar, 1 mm).

Thin myocardial sheets were mounted on glass cover slips, thawed, and placed inside an infrared laser cutter to generate precise computer-designed shapes. Scaffolds could be rotated prior to cutting in order to control the orientation of the naturally occurring tissue fibers relative to the designed shape. In typical experiments, the sheets were rotated such that the fibers were oriented parallel to the longitudinal axis of the cut pattern. A commercial 35-watt CO_2_ laser cutter was used to cut tissues into computer-designed shapes. Outlines were traced twice with 25% power and 100% speed to complete the full cut. The central portion of the scaffold, where cells were ultimately deposited, was a rectangular dog-bone shape of 5 mm wide and 6 mm long (Fig. 2a).

### Decellularization

To protect the tissue during decellularization steps, it was temporarily mounted onto removable posts positioned in the culture frame (visible in Fig. 2b). Endogenous cellular contents of the pig myocardium were then removed through a decellularization procedure as described previously^16^, with some modifications (Fig. 2c, Supplementary Methods). After decellularization, each end of the scaffold was secured to a clip in the tissue culture cassette by wedging the ends of the decellularized myocardium into a narrow slit located at the clip’s center (Fig. 1c). The tissue was mounted into the slit by a thin rectangular tab made of polyester sheeting. Friction between the tab and the sides of the slit kept the scaffold gently but securely clamped throughout the culture period and during mechanical testing. The frame ensured that clips were held at a specified length during culture.

### Cell derivation/preparation of cardiomyocytes

Complete details of cell derivation, differentiation, and preparation are found in the Supplemental Methods. The neonatal rat ventricular myocytes (NRVMs) used in most experiments were obtained using standard methods. H9 human embryonic stem cells (hESCs)^17^,^18^ were also used in some tests. To assess the ability of our constructs to characterize patient-derived cardiomyocytes, we created a new induced pluripotent stem cell (hiPSC) line from a healthy human donor using a method previously reported^19^

### Seeding of EHTs in PDMS baths

Cellular seeding was performed in customized seeding baths that confined the cell suspension to a volume directly surrounding the mounted scaffold. Scaffolds were placed face-down to increase seeding efficiency and reduce overall seeding volume (Fig. 1a). Seeding baths were made out of polydimethylsiloxane (PDMS). Elastomer and curing agent were mixed at a ratio of 10:1, poured into a 3D printed mold, and baked at 60 °C for 8 h.

Exact seeding conditions differed slightly with altered media conditions for NRVMs, hESC-CMs and hiPSC-CMs. For NRVMs/hESC-CMs individual decellularized scaffolds were seeded with 100 μL cell suspensions containing 10 million cells/ml (~1 M cells of either NRVMs, hESC-CMs/EHT). After 2 hours, 1 ml of NRVM/hESC-CM culture media consisting of high glucose DMEM with 10% horse serum, 2% FBS, 10 μg/mL insulin, 50 μg/mL ascorbic acid and 1% of antibiotic-antimycotic solution was added. Unless stated otherwise, the antibiotic-antimycotic solution was only used for the first 24 hours and removed thereafter to minimize the impact of streptomycin on stretch-activated ion channels and prevent toxicity^20^. Constructs seeded with NRVMs were treated with 100 μM bromodeoxyuridine for the first 48 hours to reduce non-cardiomyocyte cell growth^21^. EHTs seeded with iPSC-CM were also seeded with 100 μL cell suspensions containing 10 million cells/ml in seeding media (D-MEM (high glucose), 10% fetal bovine serum (FBS), 0.1 mM MEM Non- Essential Amino Acids, 2 mM L-glutamine, 5 μM of the inhibitor Y-27632 (Calbiochem)). After 2 hours, 1 ml of seeding media was added. The next day media was replaced with RPMI/B27 (with insulin).

Two days post-seeding, EHTs were removed from PDMS seeding baths and flipped so that the tissue was face-up and not in contact with culture plates to avoid cellular adhesion. Culture media was replaced every 48 hours and EHTs were cultured for up to 23 days. Following recently optimized media conditions^22^, iPSC-CM EHTs were cultured in RPMI/B27 (with insulin) and 100 ng/ml Long R3 IGF-1, 1 mM dexamethasone and 100 nM thyroid hormone (T3).

### Optical Coherence Tomography (OCT) to measure EHT dimensions

**Figure 3:**
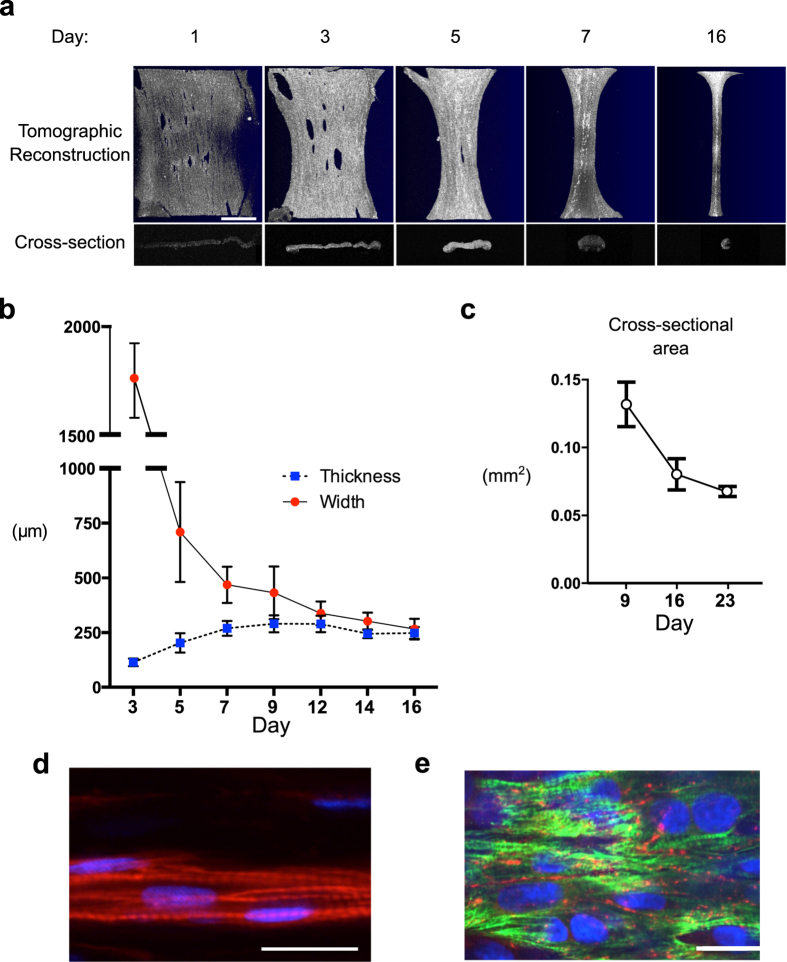
Matrix Remodeling and Histological Evaluation. (**a**) Optical coherence tomography (OCT) showing 3D reconstructions of EHTs (first row) formed by stacking serial cross-sectional images (second row) (scale bar, 1 mm). (**b**) OCT was used to follow EHT thickness and width between 3 and 16 days post-seeding. (**c**) Mean cross-sectional area assessed in 9-, 16-, and 23-day old EHTs. (**d**) Fluorescent staining for actin filaments (phalloidin, red) and nuclei (DAPI, blue) show the formation of NRVMs into rod-shaped cells within the scaffold after 16 days of culture. Striations indicate the formation of sarcomeres (scale bar, 25 μm). (**e**) Staining for cardiac Troponin T (green) and Connexin 43 (red) of an EHT seeded with human embryonic stem cell-derived cardiomyocytes. Note the formation of gap junctions between neighboring cardiomyocytes after 16 days in culture (scale bar, 25 μm).

To calculate the stress developed by each EHT, an estimation of the cross-sectional area of each construct is necessary. Cross-sectional area, width, and thickness were measured using optical coherence tomography (Fig. 3a–c). We determined the index of refraction to be 1.38. In every EHT, five evenly spaced images were taken, and the cross-sectional area of each was determined using ImageJ (National Institutes of Health). The five values were averaged to give the mean cross-sectional area. 3D reconstructions of the overall EHT were done using ThorImage OCT software (Thorlabs).

### Mechanical and functional testing of EHTs

To determine twitch force development and kinetics, EHTs were measured as early as 5 days after seeding. Clips holding the EHT were ejected from the frame and picked up by motorized micromanipulators with claw-like extensions, leaving the muscle suspended between an anchoring attachment claw on one end and a force transducer mounted to a second claw on the other (Fig. 1d). The EHT was immersed in a temperature-controlled perfusion bath equipped with electrodes for field stimulus. Throughout measurements, scaffolds were perfused with freshly oxygenated Tyrode’s solution (in mM: NaCl 140, KCl 5.4, MgCl_2_ 1, HEPES 25, glucose 10, and CaCl 1.8; pH adjusted to 7.35). Used Tyrode’s solution was collected in a sealed chamber during testing and checked to ensure adequate consistency of pH. The maximum fluctuation never exceeded 0.05 pH units. All measurements were performed at 35 °C. During biomechanical testing, NRVM - EHTs were electrically paced (12 V, 10 ms pulse width at 0.5 Hz) and the resulting twitch force was recorded (Fig. 1e, Supplementary Video 1). Human EHTs were paced at 1 Hz. Intracellular calcium concentrations were measured in some constructs using the ratiometric fluorescent indicator Fura-2 AM (see Supplementary Methods). Experiments assessing the response of EHTs to β-adrenergic stimulation are described in the Supplementary Methods.

**Figure 4:**
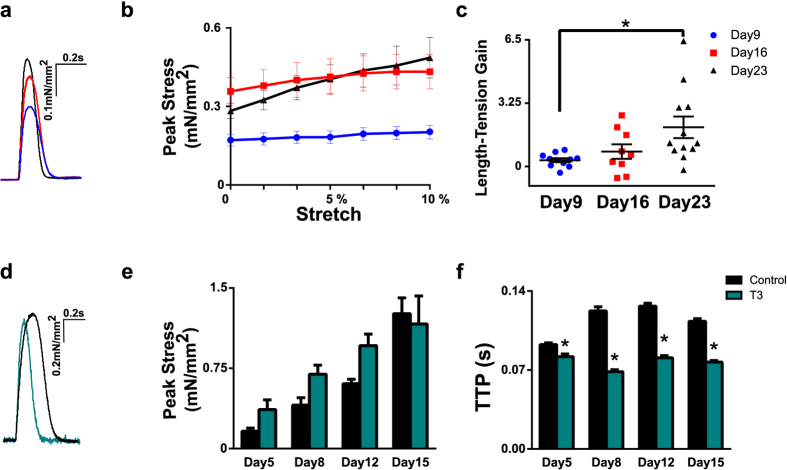
Functional assessment of EHTs. Measurements of physiological function in laser-cut EHTs. All results were obtained from NRVMs seeded into laser-cut decellularized porcine myocardium. (**a**) Representative twitch stress records after culture for 9 (blue, n = 11), 16 (red, n = 8), and 23 days (black, n = 12). (**b**) The effect of culture time and uniaxial stretch on twitch stress. (**c**) The effect of culture time on the slope of the length-stress relationship in EHTs (increase in mN/mm^2^ per unit engineering strain ANOVA, Tukey post hoc, *p < 0.05). (**d**) Representative twitch stress records from 15 day-old EHTs cultured in control media (black) or in media containing thyroid hormone T3 (teal). (**e**) The effect of T3 treatment (treated n = 10 vs. non-treated n = 12) on peak stress through repeated measurements of EHTs after 5, 8, 12, and 15 days in culture. (**f**) The effect of T3 treatment on time elapsed between stimulus to peak stress (Time-to-peak, TTP, repeated measures ANOVA,*p < 0.01).

During mechanical testing, EHTs were stretched until reaching their culture length (6 mm) and again after applying 0.1 mm incremental increases until reaching a length of 6.6 mm (representing 10% total stretch). Ten contraction events were recorded and averaged at each level of stretch. The peak stress (mN/mm^2^) was computed offline by dividing measured force by the cross-sectional area of the EHT. Characteristics of contraction kinetics were also computed offline, including the time to peak stress (TTP) and time from peak stress to 50% relaxation (RT50). The length-stress gain during isometric stretch (slope of the stretch-peak stress relationship, Fig. 4c) was calculated for each scaffold using engineering strain as the unit for stretch. The force-frequency relationship was determined while keeping EHTs at 10% stretch and taking records at 0.5, 1, 2, 4, 6, and 8 Hz. During force-frequency measurements, pacing capture of EHTs was ensured by checking real time oscilloscope readings of the force transducer voltage output.

### Shear loading and gene expression responses in EHTs

Two days after seeding, EHTs were exposed to 10% static shear or 10% stretch loading for 2 hours. This was achieved by removing clipped EHTs from standard culture frames and placing them back into either original or in specially designed shear or stretch frames. When inserted into the shear frame, both clips were forced to rotate by 7.5° about their geometric center. This produced a two-dimensional deformation in the plane of the ribbon-like EHT that equated to a shear strain component of 0.1 (10% shear strain) relative to the fiber direction. Strain in the direction of the fibers remained zero; the fiber length was not changed, therefore the matrix fibers were pulled (sheared) past each other when EHTs were mounted in this configuration. Relative changes in gene expression were quantified using Real Time-quantitative PCR (Supplementary Methods). Brain natriuretic peptide (BNP), a transcript belonging to the fetal or hypertrophic gene program was quantified relative to the housekeeping gene glyceraldehyde 3-phosphate dehydrogenase (GAPDH).

### Statistical analysis

Comparisons involving two groups were made using Student’s t-test (unpaired), whereas multiple comparisons between groups were performed by one- or two-way analysis of variance (ANOVA) with the Tukey post hoc test as indicated. The threshold for statistical significance was set at p < 0.05. Means are reported with SEM unless stated otherwise.

## Results

### Spontaneous beating and remodeling of EHTs

Testing and characterization of laser-cut decellularized myocardial scaffolds was performed using NRVMs as a cell source (unless otherwise noted). Regions of spontaneous beating within the constructs were observed two days post-seeding, transitioning to synchronously contracting constructs at approximately day five (Supplementary Video 2). EHT morphology and remodeling were followed over time using OCT imaging (Fig. 3a). Measurements in 9-, 16-, and 23-day-old EHTs show a reduction in cross-sectional area of the tissue with age (Fig. 3a,c). More detailed tracking of EHT dimensions from day 3 to day 16 shows that remodeling starts immediately, with thickness of the tissue increasing until day 9. The EHTs acquired an hourglass shape and circular cross-section of approximately 250 μm in diameter by day 16 (Figs 2d and 3b, Supplementary Video 3). Matrix compaction and tissue remodeling slowed down significantly around day 12 (Fig. 3b).

### Histological analysis confirms maturation of cardiac components

Histological analysis of NRVM EHTs at day 16 showed that cells in the EHT were distributed evenly throughout the constructs (Supplementary Fig. 1). Furthermore, sections stained for myofilament proteins exhibited a striated pattern that indicated the presence of organized sarcomeres (Fig. 3d). Fluorescent staining for connexin 43 in EHTs seeded with hESC-derived cardiomyocytes showed that the cells had also formed gap junctions, indicating electrical coupling of individual cardiomyocytes (Fig. 3e).

### Time in culture improves cardiac maturation

A series of experiments were performed to demonstrate the potential of this system for biomechanical and functional characterization of engineered myocardium. First, to determine the effects of culture time on stress development and twitch kinetics, scaffolds were tested after either 9, 16 or 23 days in culture (n = 11, 8 and 12 respectively; Fig. 4a–c). Time in culture significantly impacted EHT peak twitch stress (one-way ANOVA, p < 0.05). Between day 9 and day 23, peak stress increased 2.4 fold (from 0.20 ± 0.03 to 0.49 ± 0.08 mN/mm^2^, Fig. 4b). Furthermore the sensitivity of peak stress to stretch (Frank-Starling response) increased significantly with time in culture (length-stress gain, p < 0.01, Fig. 4c). Twitch kinetics, as measured by time to peak stress (TTP) and time from peak stress to 50% relaxation (RT50), did not change significantly with culture time (Supplementary Fig. 2a). We also observed a negative force-frequency response (Supplementary Fig. 2b).

### Thyroid hormone speeds contractile kinetics

To assess the sensitivity of our system towards changes in contractility and twitch kinetics, we subjected EHTs to a pharmacological perturbation known to alter the intrinsic properties of cardiac muscle. In rodents, an increase in thyroid hormone (T3) promotes expression of the faster α isoform of myosin heavy chain^23^. Hence, EHTs were treated in culture for 15 days with T3 (10 ng/ml, Fig. 4d–f) and tested repeatedly in a longitudinal study. EHTs were characterized functionally and then returned to culture conditions at days 5, 8, 12, and 15. T3 had no significant effect on the evolution of peak stress development across time points (2-way repeated measures ANOVA) (Fig. 4e). However, T3 treatment significantly accelerated rates of contraction and relaxation at every time point analyzed in comparison to the non treated control (T3 treatment n = 10, no treatment n = 12; p < 0.001, Fig. 4f and Supplementary Fig. 3). After 15 days in culture, EHTs treated with T3 had a TTP of 77 ± 2 ms in comparison to 113 ± 6 ms and a RT50 of 63 ± 3 ms versus 130 ± 3 ms in untreated samples.

### NRVMs produce less force when grown in scaffolds with transversely oriented fibers

**Figure 5:**
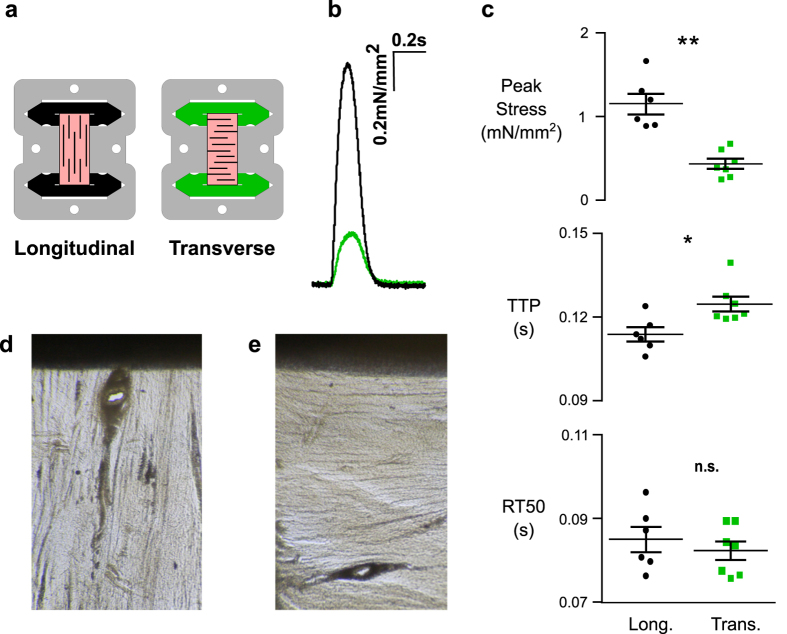
Anisotropic Twitch behavior. (**a**) Schematic illustrating longitudinal (black) vs. transverse (green) fiber alignment of tissue (pink). (**b**) Representative twitch stress records of EHTs from longitudinal (n = 6) or transverse (n = 7) fiber alignments after 9 days in culture. (**c**) The effect of fiber orientation on peak stress (Student’s t-test, **p < 0.001), on time elapsed between stimulus to peak stress (Time-to-peak, TTP, Student’s t-test, *p < 0.05) and on time elapsed between peak stress to 50% peak stress (Relaxation-time 50, RT50, n.s.). Decellularized Scaffold under light microscope showing longitudonal (**d**) and transverse (**e**) fiber alignment.

The fiber direction in native cardiac tissue is visible with the naked eye (Fig. 5d,e). A major advantage of scaffolds laser-cut from native myocardium is the ability to easily control fiber orientation of the matrix material. To determine whether embedded cells would follow the alignment cues provided by the matrix of the EHT, tissues were cut such that the native fiber angle was oriented either parallel to the long axis of the construct (longitudinal, Fig. 5a,d) or 90 degrees relative to the long axis (transverse, Fig. 5a,e). Longitudinal and transverse scaffolds were seeded and cultured for 6 days prior to biomechanical characterization. When function was assessed at 10% stretch, longitudinal scaffolds (n = 6) produced on average a peak stress that was 240% greater than transverse controls (n = 7) under the same conditions (p < 0.001, Fig. 5b,c). TTP was 11% longer in transverse EHTs (p < 0.05, Fig. 5c), while no difference in the rate of relaxation was observed. To further assess whether the natural structure, biochemical composition, and density of the native scaffold provide unique advantages to EHT function, we also compared EHTs made from laser-cut decellularized myocardium with tissues made from anisotropic electrospun gelatin scaffolds (Supplementary Fig. 4). To facilitate comparison, both scaffold materials were coated with fibronectin prior to cell seeding. At day 9 post-seeding, both tissues exhibited a positive Frank- Starling response. However, decellularized myocardial scaffolds produced significantly higher peak twitch stresses (Supplementary Fig. 4).

### BNP expression is stimulated by fiber stretch but not shearing stretch

**Figure 6:**
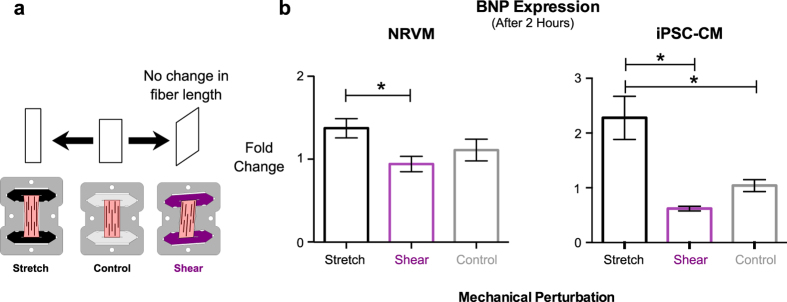
Opposing effects of static shear and stretch loading. (**a**) Schematic illustrating normal 10% stretch (black, n = 18 (NRVM) & n = 9 (iPSC-CM)), 10% shear stretch (purple, n = 22 (NRVM) & n = 4 (iPSC-CM)) or no stretch (grey, n = 16 (NRVM) & n = 8 (iPSC-CM))) applied to EHTs and (**b**) the effect of 2 hours of these regimes on brain natriuretic peptide (BNP) expression (normalized to GAPDH, 1-way ANOVA, *p < 0.05).

Laser-cut EHTs with controlled fiber orientations enable the study of novel mechanical perturbations and the cellular signaling cascades they may activate. Responses of the tissue to shear strain in an vitro system is of particular interest since the ventricular myocardium is exposed to shear loading throughout the cardiac cycle^24^, which varies according to myocardial transmural depth^25^. Loading regimes *in vivo* are complex and contain varying shear and stretch components making it difficult to separate the individual responses. Our *in vitro* system allows the application of unambigously defined mechanical loads, including the ability to apply pure stretch and shear separately. To determine the effects of shear loading on cardiac gene expression, we exposed two-day old NRVM EHTs to static 10% stretch applied for two hours, either along the fiber direction (n = 18), at an angle such that fibers were sheared past each other while remaining at roughly constant length (n = 22), or as a control at original culture length (n = 16) (Fig. 6a). The two-day-old initial time point was chosen because EHTs at this stage have remodeled minimally, and their ribbon-like shape allows the clean application of a shearing stretch. We found that after two hours, fiber stretch and shear stretch had elicited significantly different responses with regard to BNP expression (p < 0.05, Fig. 6b). As expected, fiber stretch led to increased BNP expression, but surprisingly no expression change in the sheared tissues with respect to non-stretched control was observed. The differential response to stretch vs. shear loading was confirmed in separate experiments using EHTs formed with cardiomyocytes derived from human induced pluripotent stem cells (hiPSC-CMs, Fig. 6b). Detailed fold-change values can be found in Supplementary Table 1.

### Laser-cut decellularized myocardium can be used for biomechanical characterization of human iPSC-derived cardiomyocytes

**Figure 7:**
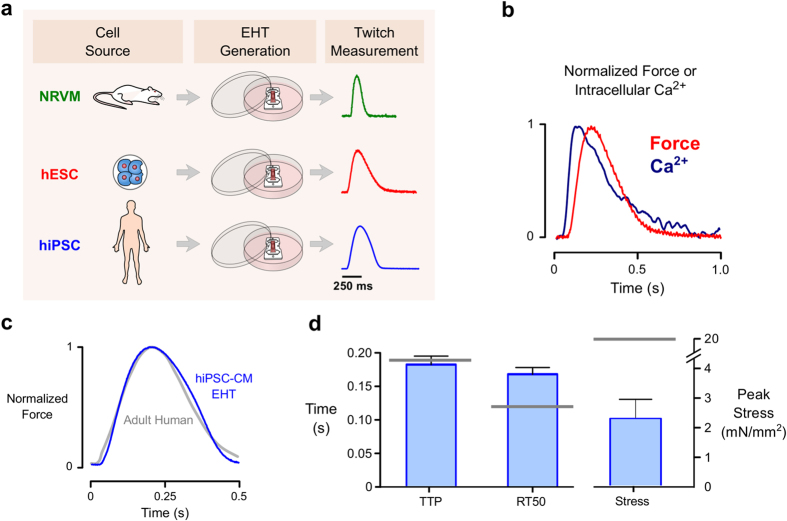
EHTs seeded with human-derived cardiomyocytes. (**a**) Laser-cut decellularized myocardium serves as a viable scaffold for several cardiomyocyte sources, including neonatal rats, human embryonic stem cells (hESCs), and human induced pluripotent stem cells (hiPSCs). (**b**) A representative trace showing simultaneous acquisition of intracellular Ca^2+^ (fura-2 fluorescence) and isometric twitch tension measured in an EHT containing hESC-derived cardiomyocytes. (**c**) A representative twitch trace recorded in an EHT containing hiPSC-derived cardiomyocytes. The twitch is superimposed on a twitch produced by a human right ventricular trabecula, digitized from Nejad *et al.*^57^ (**d**) Mean twitch kinetic parameters and peak tension measured in hiPSC-EHTs (n = 8). Gray bars indicate mean properties reported for human left ventricular muscle strips by Mulieri *et al.*^58^. Abbreviations: TTP, time to peak stress; RT50, time from peak to 50% relaxation.

To determine whether the method could be readily applied to human-derived cells, we also seeded decellularized scaffolds with cardiomyocytes derived from human embryonic cells (hESC-CM) or hiPSC-CMs (Fig. 7a). Using standard media conditions, hESC-CMs produced beating scaffolds with measurable intracellular Ca^2+^ transients and maximum twitch stress of 1.7 mN/mm^2^ at day 16 (n = 3) (Fig. 7b). Scaffolds seeded with hiPSC-CM (obtained from a healthy donor) were cultured in media containing thyroid hormone, IGF-1, and the glucocorticoid analog dexamethasone, factors recently shown to promote cardiomyocyte maturation^22^. hiPSC-CM EHTs produced an average peak stress (force divided by cross-sectional area) of 2.2 ± 0.76 mN/mm^2^ at 8% stretch, with maxium peak stress of 6.5 mN/mm^2^ (n = 8, Fig. 7d). The magnitude of raw force, for purposes of comparison, was an average of 0.3 ± 0.09 mN. One of the hiPSC-CM EHTs achieved a maximum peak force of 0.86 mN. Asessing the kinetics of these constructs showed a RT50 of 168 ± 10 ms and a TTP of 181 ± 12 ms (at a 1 Hz pacing frequency). hiPSC-CM EHTs also responded to the β-adrenergic agonist Isoproterenol by showing appropriate lusitropic behavior (shorter TTP and RT50) (Supplementary Fig. 5).

## Discussion

The data presented here demonstrate that laser-cut decellularized myocardium can function as an effective scaffold for engineered heart tissue. This method takes advantage of the natural anisotropy of cardiac ECM while permitting creation of macroscopic customized shapes that are suitable for measurement of contractile force and other experiments. Some of these, such as the assessment of fiber shear effects on BNP production, are to the best of our knowledge unique among *in vitro* studies. Laser-cut decellularized myocardial scaffolds also proved to be versatile platforms for EHT creation, producing viable tissues from NRVMs, hESC-CMs, and hiPSC-CMs alike. This suggests that the approach could be useful in a variety of applications, including physiological characterization of patient-derived cardiomyocytes and *in vitro* studies of cardiomyocyte mechanotransduction and remodeling.

The observation that shearing stretch tended to decrease BNP expression is surprising, as it is typically seen as a stretch-induced factor. Studies have shown that biaxially stretched cardiac cells increase BNP production^26^. This coincides straightforwardly with its traditional physiological role as a volume-regulating hormone, promoting diuresis in response to excess ventricular filling^27^. In line with this concept, our constructs did increase BNP expression when subjected to fiber stretch (Fig. 6b). The shear experiment suggests an alternate mechanism that could allow BNP transcription to be triggered not just by increased preload, but by increased afterload as well. Fiber shearing during systole is a large component of normal myocardial deformation, and is observed at the organ level as ventricular torsion. It has been shown that increased afterload can reduce cardiac torsion during systole by as much as 10%^28^, which by extension may decrease fiber shear by a similar amount. If BNP transcription is enhanced by the absence of shear, as our measurements suggest, it would therefore constitute a new mechanism by which cells could sense changes in arterial loading (afterload reflex). This could be seen as being in line with recent clinical findings, where reduced left ventricular global longitudinal strain in patients with chronic heart failure was associated with increased plasma concentrations of N-terminal-pro-brain- natriuretic-peptide^29^. Similarly, such a mechanism could explain why patients with systemic hypertension show elevated BNP levels in the absence of heart failure^30^

Another novel insight provided by our technology is that neonatal or fetal-like cardiomyocytes respond more potently to local matrix cues for alignment than to macroscopic loading (Fig. 5). In systems that use isotropic gels as scaffold material, cells appear to align in the direction of greatest stiffness or applied loads^31^,^32^. Hence, to achieve anisotropy in hydrogel-based tissues, externally applied boundary loads appear to be required^32^,^33^,^34^. In our system, cells are provided with a scaffold that contains the natural fiber structure. We hypothesized that seeded cells would respond to local cues found in the preserved ECM structure, leading to anisotropic functional properties in spite of how the tissue was oriented in the tissue cassette. Indeed, we found that in transversely oriented scaffolds the muscle cells exhibited slower twitch kinetics and produced only a fraction (~30%) of the peak tension observed in longitudinally-oriented samples. It is interesting to note that similar behaviour has been shown in native cardiac muscle tissue preparations. For instance, Lin and Yin^10^ showed that barium-induced contractures of rabbit myocardium produce roughly 50% less active force in the cross-fiber direction than in the fiber direction. It therefore appears that native scaffolding guides cells to produce a realistic anisotropy in active contractile properties. When compared to anisotropic electrospun gelatin scaffolds, laser-cut decellularized myocardium showed stronger peak stresses and a tendency toward faster twitch kinetics. This suggests that the biochemical and biomechanical cues found in the decellularized native myocardium confer specific advantages to the process of *in vitro* tissue formation over and above those achieved through anisotropic synthetic scaffolds.

There is a large and diverse body of methods for the generation of engineered heart tissue, each one with benefits for specific applications^4^,^7^. In evaluating our approach, it is useful to draw some comparisions with other methods. These fall into two broad categories: macroscopic bulk tissue constructs^34^,^35^,^36^,^37^,^38^ and microfabricated cell assemblies (microtissues or ‘heart on a chip’ approaches)^39^,^40^,^41^.

Macroscopic constructs have the advantage of being large enough to produce contractions that can be measured directly with traditional force transducers. In contrast, measuring force in microtissues requires that they be attached to a deformable elastic structure of known stiffness. Substrate deformation allows the force to be inferred, but also means that substantial muscle shortening is always a component of such measurements. Twitches recorded in macroscopic preparations can be characterized under isometric conditions that essentially eliminate length dependent effects and provide high fidelity, high-resolution characterizations of active stress generation.

Microtissues on the other hand require fewer cells, have smaller dimensions, and can achieve anisotropy by direct micropatterned cues. The small form factor can be a key consideration due to the oxygen diffusion limit for physiological tissue. Constructs thicker than ~200 μm that are seeded with physiological cell densities can exhibit poor cell viability due to nutrient and oxygen exchange problems at their core^42^. Cardiac microtissues typically have a cross-sectional area of 0.01 mm^2^ with a diameter not exceeding 70 μm, the equivalent to just a few cells^40^. This is far thinner than typical fibrin- or hydrogel-based EHTs, where the average diameter is ~800 μm^43^ and cross-sectional areas are typically ~1 mm^2 ^44. Beyond the diffusion advantage, some microtissues are grown on patterned anisotropic substrates that impart improved Ca^2+^ handling, excitation-contraction coupling, alignment, and peak force^45^,^46^.

The laser-cut decellularized matrix approach presented here can be seen as a compromise between macroscopic and microfabricated EHTs – our constructs are large enough to have robust contractile forces while possessing a thin, realistically anisotropic form. By starting with native myocardium, thin but structurally stable anisotropic scaffolds can be cut. After 16 days in culture, EHTs had an average diameter of 250 μm and cross-sectional area of ~0.1 mm^2^, substantially smaller than typical macroscopic constructs. Peak stresses produced by our NRVM EHTs were ~1 mN/mm^2^ on average and reached 3 mN/mm^2^ in some cases. These values are directly comparable to others in the field, who reported average stress within the 0.1 - 4 mN/mm^2^ range, with the highest reported stress to date being 9 mN/mm^2 ^4,47. As a point of reference, isolated neonatal rat trabeculae show peak stress of around 8–9 mN/mm^2 ^48 and thin adult rat trabeculae of about 42 mN/mm^2 ^49. In the case of our iPSC-CM EHTs peak stresses averaged to ~2.2 mN/mm^2^ putting them within commonly reported values^50^,^51^,^52^ but below intact left ventricular muscle strips (Fig. 7d).

Although improvements in peak stress are still needed, it is interesting to note that the NRVM EHTs described here are nonetheless approaching near-native twitch kinetics. In 15 day old EHTs after T3 treatment, the measured RT50 of around 59 and TTP of 83 ms (2 Hz) are similar to those reported for thin native rat trabeculae (45 and 75 ms respectively at a pacing frequency of 2 Hz^49^). A comparison of twitch kinetics among different EHT techniques would also be informative. However, this is currently not possible because of the wide range of metrics used to report twitch kinetics in EHTs^5^. The only other study to report both TTP and RT50 in NRVM based 2 week old EHTs was the work of Morgan *et al.*^53^. Their reported RT50 and TTP values (125 and 150 ms respectively at 0.5 Hz pacing)^53^ are notably slower than those observed in our method. This difference could potentially be explained by our T3 media substitution^54^.

Ensuring that EHTs recapitulate adult myocardial function as closely as possible remains a primary concern as the field seeks to use human engineered constructs for preclinical drug screening and patient-specific disease modeling^38^,^41^,^55^. For instance, it is hoped that EHTs seeded with cells from patients with cardiomyopathy will lend insights into disease mechanisms. However, animal studies have already demonstrated that the effects of sarcomeric mutations depend on the isoform profile of other proteins in the sarcomere^56^, something that varies substantially during maturation^5^. One way to gauge the overall maturation of EHTs is to perform detailed characterisation of their twitch kinetics^44^. Using an optimized media formulation recently reported by another group^22^, we were able to create human EHTs with twitch morphology essentially equivalent to human right ventricular trabeculae^57^ (see Fig. 7c). Left ventricular muscle strips seem to possess slightly faster kinetics^58^, but hiPSC-CM EHTs are beginning to approach even these values (Fig. 7d). Maturation in our constructs could be further improved by applying an electrical pacing regime in culture just above the intrinsic beating frequency^59^. Based on the experience of Godier-Furnémont *et al.*^59^, we anticipate that pacing during culture would address the lack of a positive force-frequency relationship currently seen in our rat and human EHTs (Supplementary Fig. 2).

In conclusion, our method for generating artificial cardiac tissue joins several others that have been developed in recent years (reviewed elsewhere^4^,^7^). Evaluating the utility of any given approach depends as much on the intended use of the EHT as it does on any particular metric of performance. In addition to standard isometric twitch behavior, which is extremely robust in our constructs, our approach makes possible for the first time new biomechanical assays in which properties of the tissue can be assessed in relation to realistic structural and functional anisotropy. As such, we believe it provides a platform for mechanotransduction studies that are not currently served by any other approach. Although we have focused on decellularized native myocardial scaffolds, the tissue culture cassette system was easily adapted for use with synthetic laser-cut electropsun scaffolds (Supplementary Fig. 4), demonstrating that the overall cassette approach could be adapted to accommodate a wide variety of scaffold types. Because the system can be used to form functional tissue from cultured human embryonic as well as induced pluripotent - stem cell derived cardiomyocytes, we anticipate that it will ultimately find use in patient-specific *in vitro* modeling. For inherited disorders such as familial hypertrophic cardiomyopathy, where tissue remodeling, mechanotransduction, and contractile mechanics play prominent roles, we believe this platform is a suitable alternative to current methods.

## Additional Information

**How to cite this article**: Schwan, J. *et al.* Anisotropic engineered heart tissue made from laser-cut decellularized myocardium. *Sci. Rep.* **6**, 32068; doi: 10.1038/srep32068 (2016).

## Supplementary Material

Supplementary Information
