# Supplementary material for: Anisotropic engineered heart tissue made from laser-cut decellularized myocardium
Source: Sci Rep. 2016 Aug 30;6:32068. doi: 10.1038/srep32068 (PMC5004193; doi:10.1038/srep32068)
Supplement: Supplementary Information [file srep32068-s1.pdf]

SUPPLEMENTARY MATERIAL

Anisotropic engineered heart tissue made from laser-cut decellularized myocardium

Jonas Schwan, MS[1], Andrea T. Kwaczala, PhD[1,2], Thomas J. Ryan[1], Oscar Bartulos, PhD[3,4], Yongming Ren, PhD[3,4], Lorenzo R. Sewanan[1], Aaron H. Morris, MS[1,6], Daniel L. Jacoby, MD[3], Yibing Qyang, PhD[3,4,5,6], and Stuart G. Campbell, PhD*[1,7]

[1] Department of Biomedical Engineering, Yale University, New Haven, CT 06510, USA.
[2] Department of Biomedical Engineering, University of Hartford, West Hartford, CT 06117, USA.
[3] Yale Cardiovascular Research Center, Section of Cardiovascular Medicine, Department of Internal Medicine, Yale School of Medicine, New Haven, CT 06510, USA
[4] Yale Stem Cell Center, Yale University, New Haven, CT 06510, USA
[5] Department of Pathology, Yale University, New Haven, CT 06510, USA
[6] Vascular Biology and Therapeutics Program, Yale School of Medicine, New Haven, CT 06510, USA
[7] Department of Cellular and Molecular Physiology, Yale School of Medicine, New Haven, CT 06510, USA

Supplementary Methods

Decellularization

All incubations were performed at room temperature unless stated otherwise. In brief, the tissues were incubated in a cell lysis buffer (10 mM Tris, 0.1% wt/vol EDTA, pH 7.4) for 2 hours followed by a 40-minute incubation in sodium dodecyl sulfate (0.5% wt/vol in PBS) with gentle agitation (35 rpm on an incubated shaker). Tissues were washed three times in PBS and incubated in DMEM containing 10% FBS for 24h at 37°C to remove residual DNA[1]. Sterilization was performed by placing the tissues in 0.1% peracetic acid and 4% ethanol for 5 minutes. Tissues were washed three times in PBS before being attached to PTFE clips for seeding and culture.

Cell derivation/preparation of cardiomyocytes

Neonatal rat ventricular myocytes (NRVM)
NRVMs were isolated from 1-3 day old Sprague Dawley rat pups using a standard protocol[2]. In brief, hearts were dissected, minced, and digested using a solution containing Type II collagenase (0.5mg/ml, Worthington Biochemical Corporation) and protease (0.1mg/ml, Sigma Aldrich). During constant rotational digestion, cell suspensions were collected in batches every 20-30 minutes. Each aliquot was spun by gentle centrifugation and pre-plated for 90 minutes on standard cell-culture plates in DMEM containing 10% FBS and Pen/Strep. The remaining cell suspension of cardiomyocytes, considered fibroblast-depleted, was collected from the pre-plating culture plates, combined into a single aliquot, spun down and resuspended in complete culture media for seeding into EHTs. 1 million cells were seeded per decellularized scaffold.

Human embryonic stem cell (hESC) culture and cardiac differentiation
hESC from the hESC line H9[3] were cultured with mTeSR (Stemcell Technologies) on reduced growth factor- Matrigel (BD Falcon)-coated plates until they reached 80-90% confluence. Cells were detached with 1mg/ml Accutase (Innovative Cell Technologies) for 5 minutes and $5x10^4$ cells/cm^2 were plated on Matrigel-coated 12-well plates in the presence of 5µM of the inhibitor Y-27632 (Calbiochem), which was removed the following morning. Cells were maintained in mTeSR until they had 80-85% confluence (4 days). Differentiation to cardiomyocytes was performed as previously described[4]. Briefly, four days after plating, hESCs were cultured in B27/RPMI medium; RPMI 1640 (Life Technologies) with B27 minus insulin (Life Technologies), containing 10µM CHIR99021 (Selleck Chemicals). Twenty-four hours later (day 1 of differentiation), the medium was replaced with fresh B27/RPMI medium. On day 3 of differentiation half of the medium was removed and the other half was mixed with fresh B27/RPMI medium and 5µM of the Wnt Inhibitor IWP4 (Stemgent) was included in the solution. On day 5 of differentiation, the medium was replaced with fresh B27/RPMI medium. After day 5, medium was replaced every 3 days with fresh B27/RPMI medium. On day 14 of differentiation, cardiomyocytes were detached with 1mg/ml Accutase for 10 minutes and a single cell suspension was obtained after application of mechanical force. Only batches with 45-75% cardiomyocytes were utilized for experiments (based on cardiac troponin T staining of a test aliquot). Spheroids were prepared with $1x10^4$ cells per spheroid in 0.24% methylcellulose (Sigma-Aldrich) as previously described[5], with some modifications. Twenty-four hours after plating the cells on 96-U bottom low attachment plates, the vast majority of the cells

were aggregated into spheroids. Spheroids were collected and washed 3 times with RPMI 1640. One million cells (~200 spheroids) were seeded per decellularized scaffold.

iPS cell derivation

Briefly, we collected 10 ml of whole blood from a healthy, male donor by venipuncture under the approval of Yale Institutional Review Board. Peripheral blood mononuclear cell (PBMCs) were isolated using a Ficoll-paque (GE healthcare) based method. PBMCs were cultured on anti-CD3 antibody (BD Biosciences) coated plate in 10% FBS RPMI-1640 medium with the addition 120 ng/ml of IL2 (BD Biosciences) for 5 days. To enable reprogramming of PBMCs to iPSC, PBMCs were infected with Sedai virus (CytoTune®-iPS Sendai Reprogramming Kits, Invitrogen) according to the manufactory instruction. The infected cells were seeded on irradiated mouse embryonic fibroblast cell covered plates for 3-4 weeks in human embryonic stem cells medium (20% Knock out Serum Replacement,1% Glucose,1% NEAA,55uM beta-mecaptoethanol in DMEM/F12 basal medium containing bFGF 10ng/ml). The clones were then picked and expanded for differentiation after 15 passages to ensure removal of virus and stained for pluripotency markers OCT4, SSEA-4, NANOG, TRA-1-60, and alkaline phosphatase (**Supplemental Fig. 6)**.

Cardiac Differentiation of iPSCs

Briefly, iPSCs were dissociated with dispase and gentle agitation and transferred to a 15 ml conical tube. After settling for 7 minutes, the supernatant was removed and the pellet resuspended in 2 ml mTeSR media (StemCell Technologies) and transferred to a Matrigel-coated plate (BD Biosciences). Cells were maintained in mTeSR media until they reached 90% confluency (4-5 days) Once confluency was reached, iPSCs were cultured in B27/RPMI medium containing 25ng/ml CHIR99021 (Selleck Chemicals). Twenty-four hours later (day 1 of differentiation), the medium was replaced with fresh B27/RPMI medium. On day 3 of differentiation half of the medium was removed and the other half was mixed with fresh B27/RPMI medium and 5µM of the Wnt Inhibitor IWP4 (Stemgent). On day 5 of differentiation, the medium was replaced with fresh B27/RPMI medium. After day 5, medium was replaced every 3 days with fresh B27/RPMI medium. Beating clusters were usually observed at day 8-9. At day 14, cells were subjected to dissociation.

Electrospun Gelatin Scaffolds

The electrospinning setup was similar to that described previously [6]. Briefly, an 18g needle was set up at a distance of 15 cm from the center of a rotating cylindrical collector approximately 15 cm in diameter. The electrospinning solution was derived from that prepared previously[7]. 10g of Gelatin (Type B from porcine skin, Sigma) was dissolved in 42g glacial acetic acid, 21g ethyl acetate, and 10g dH2O by heating to 50C for two hours. This solution was loaded into a syringe and dispensed at 10 uL/min into a 20 kV electric field. Samples were stored at room temperature until use. Crosslinking was performed using 0.5% Glyceraldehyde in 70% Ethanol overnight as described previously[8]. For scanning electron microscopy (SEM), samples were mounted onto SEM stubs with carbon tape, coated with approximately 3 nm of irridium and imaged with a Hitachi SU-70 SEM. For EHT experiments, gelatin scaffolds were laser-cut and were placed into the tissue culture cassettes as described previously and sterilized with 70% Ethanol for one hour. Gelatin scaffolds and concomitant decellularized scaffolds were washed three times in PBS, incubated in DMEM containing 10% FBS for 24h at 37°C, and then coated overnight with 20ug/ml Fibronectin solution at 37°C before seeding.

Mechanical and functional testing of EHTs

Ratiometric fluorescent indicator Fura-2 AM
Fura-2 AM loading was achieved by incubating the EHT at room temperature in loading solution (Tyrode's supplemented with 17µg/ml Fura-2 AM, 0.2% Pluronic F127, 0.5% Cremophor EL, 4.3 µg/mL TPEN) for 20 minutes. The test apparatus was built around an inverted fluorescence microscope (Nikon Ti-U) with an attached ratiometric spectrofluorometry system (RatioMaster, PTI). A central segment of the EHT was placed above a 10X objective, and the sample was excited with a rapidly alternating sequence of 340/380 nm light. Emitted light from the specimen was filtered, and the response centered at 510 nm was recorded by the spectrofluorometer. Custom post-processing routines separated the interleaved excitation response signals and computed a final ratiometric response

Inotropic response
To test for a positive inotropic response 100 nM of isoproterenol was added to freshly bubbled Tyrode's solution, which also contained 1 mM of ascorbic acid. Tissues were equilibrated in Tyorde's solution for 30 minutes and measured at 8% stretch. Solutions were changed to isoproterenol containing Tyrode's and the tissue was equilibrated for another 30 minutes before measurements were taken again. Isoproterenol was washed out to confirm that the reversibility of the response.

Shear loading and gene expression responses in EHTs

Real-time quantitative PCR
To assess relative changes in gene expression, real-time quantitative PCR was performed EHTs were removed from the tissue culture cassette using a dissecting microscope, submerged in Trizol reagent (Invitrogen), and homogenized using a Precellys24 bead beater (Bertin Technologies). RNA was isolated using a standard procedure[9] followed by a DNAse I digestion treatment (Turbo DNA-free, Ambion). Nucleic Acid Purity was assessed using a nanoDrop spectrophotometer (Thermo Scientific). Reverse transcription (using a High capacity cDNA Reverse Transcription Kit, Applied Biosystems) was be done in the presence of RNAse inhibitor (Applied Biosystems) to synthesize high quality cDNA. Changes in BNP and GAPDH were quantified by real-time PCR using the iQ SYBR Green Supermix (Biorad) using PrimePCR primers (BioRad) for NRVM scaffolds and customized primers for human genes (as shown below). Gene expression was determined through delta-delta CT method[10].

Gene of interest	***Forward***	***Reverse***
GAPDH	TGTTGCCATCAATGACCCCTT	CTCCACGACGTACTCAGCG
BNP	ACCGCAAAATGGTCCTCTAC	GCCAGGACTTCCTCTTAATG

Histological evaluation of EHTs

For immunofluorescence, EHTs were fixed in 4% PFA while being kept at culture length in the standard culture cassettes for 2 hours at room temperature. After the fixation step, EHTs were washed in PBS twice and carefully removed from the clips using a dissecting scope. For phalloidin staining, the entire EHT sample was incubated in 1:100 Phalloidin (Alexa Fluor 568 - Life Technologies Catalog # A12380) diluted in PBST (0.1% Tween 20 in PBS) for 30 minutes, and then washed in PBS for 3 x 2 minutes. For all other stains, antigen retrieval was necessary. Fixed tissues

were incubated in a 95°C hot sodium citrate buffer (10mM Sodium Citrate, 0.05% Tween 20, pH 6.0) in a water bath at 90 °C or 20 minutes. The entire EHT sample was cooled at room temperature for 20 minutes before washes in PBST for 2 x 2 minutes. EHTs were blocked for 30 minutes in PBST containing 10% [vol/vol] goat serum. Subsequently, EHTs were incubated with 1:100 anti Connexin-43 rabbit polyclonal antibody (Catalog # C6219, Sigma-Aldrich) and 1:100 anti-Troponin T mouse monoclonal antibody (Catalog # MS295, P Fisher Scientific) for 24h at 4°C. Three washes with PBST were performed before incubating in 1:250 Alexa Flour 488 goat anti-mouse IgG (H+L, Catalog # A11029, Life technologies) and 1:250 Alexa Flour 568 goat anti- rabbit IgG (H+L, Catalog # A11036,Life technologies) for 1h at room temperature. Following secondary antibody incubation, EHTs were washed in PBS 3 x 2 minutes and coverslipped using DAPI containing fluoroshield mounting media (Catalog # F6057, Sigma-Aldrich). All imaging was performed using a Leica LSM 510 system.

Supplementary Figures

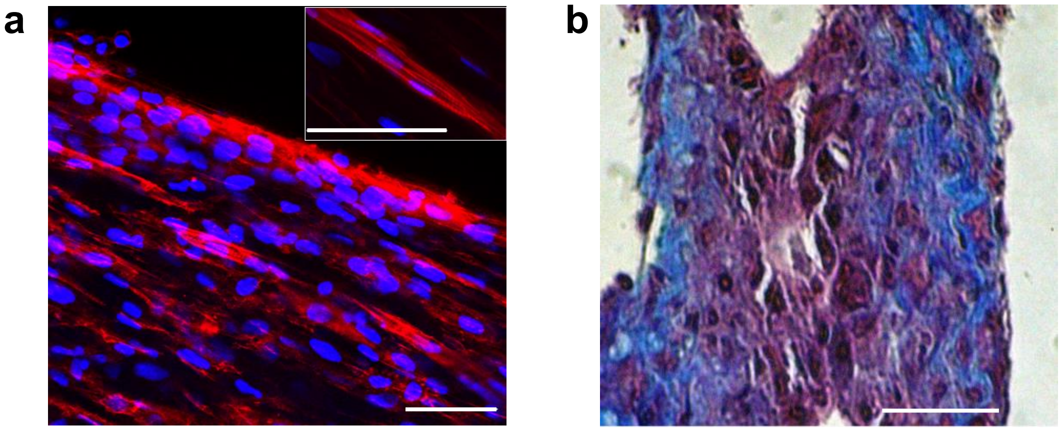

Supplementary Figure 1 | Histological Analysis
Cell distribution in the scaffold is shown using histology. All results were obtained from NRVMs seeded into laser-cut decellularized porcine myocardium at day 16. Scale bars are 50 µm (**a**) Immunofluorescent staining for actin filaments (phalloidin, red) and nuclei (DAPI, blue), Inset shows magnification illustrating striated actin structure. (**b**) Masson's-trichrome Stain of longitudinally cut EHT. Nuclei (dark brown/black) are evenly distributed.

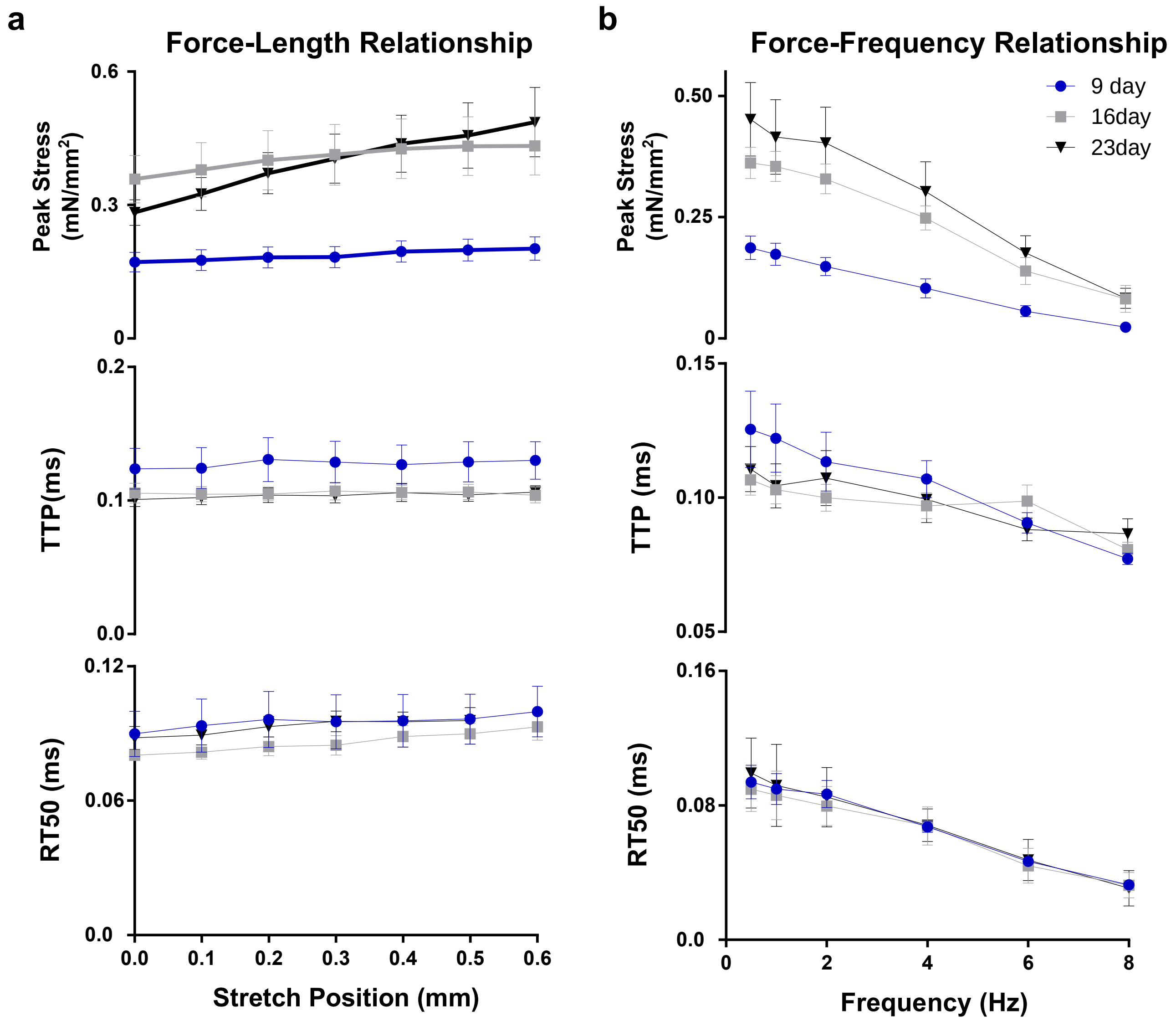

Supplementary Figure 2 │ Detailed functional behaviour of EHTs
Measurements of physiological function in laser-cut EHTs. All results were obtained from NRVMs seeded into laser-cut decellularized porcine myocardium. (**a**) Force-length relationship for peak tension (repeated for purposes of comparison from Figure 4b in the main manuscript) and twitch kinetics (time to peak and relaxation time 50) of EHTs cultured for 9 (blue, n=11), 16 (grey, n=8), and 23 days (black, n=12), culture length of EHTs are 6mm meaning that a stretch position of 0.6mm represents 10% stretch. (**b**) Force-frequency relationship for peak tension and twitch kinetics at 8% stretch.

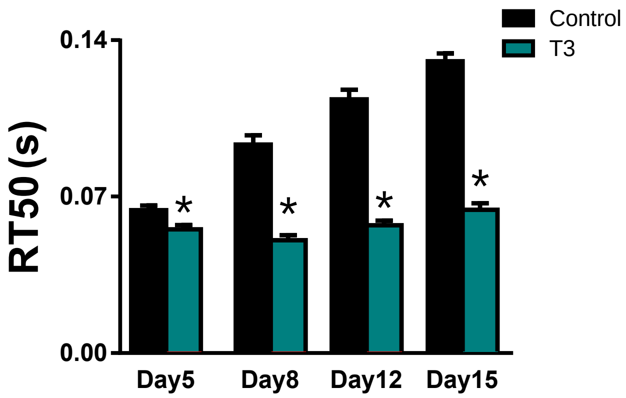

Supplementary Figure 3 | Effects of T3 on relaxation
Measurements of relaxation time (RT50) in T3 treated (teal, n=10)/non treated (black, n=12) laser-cut EHTs. All results were obtained from NRVMs seeded into laser-cut decellularized porcine myocardium.

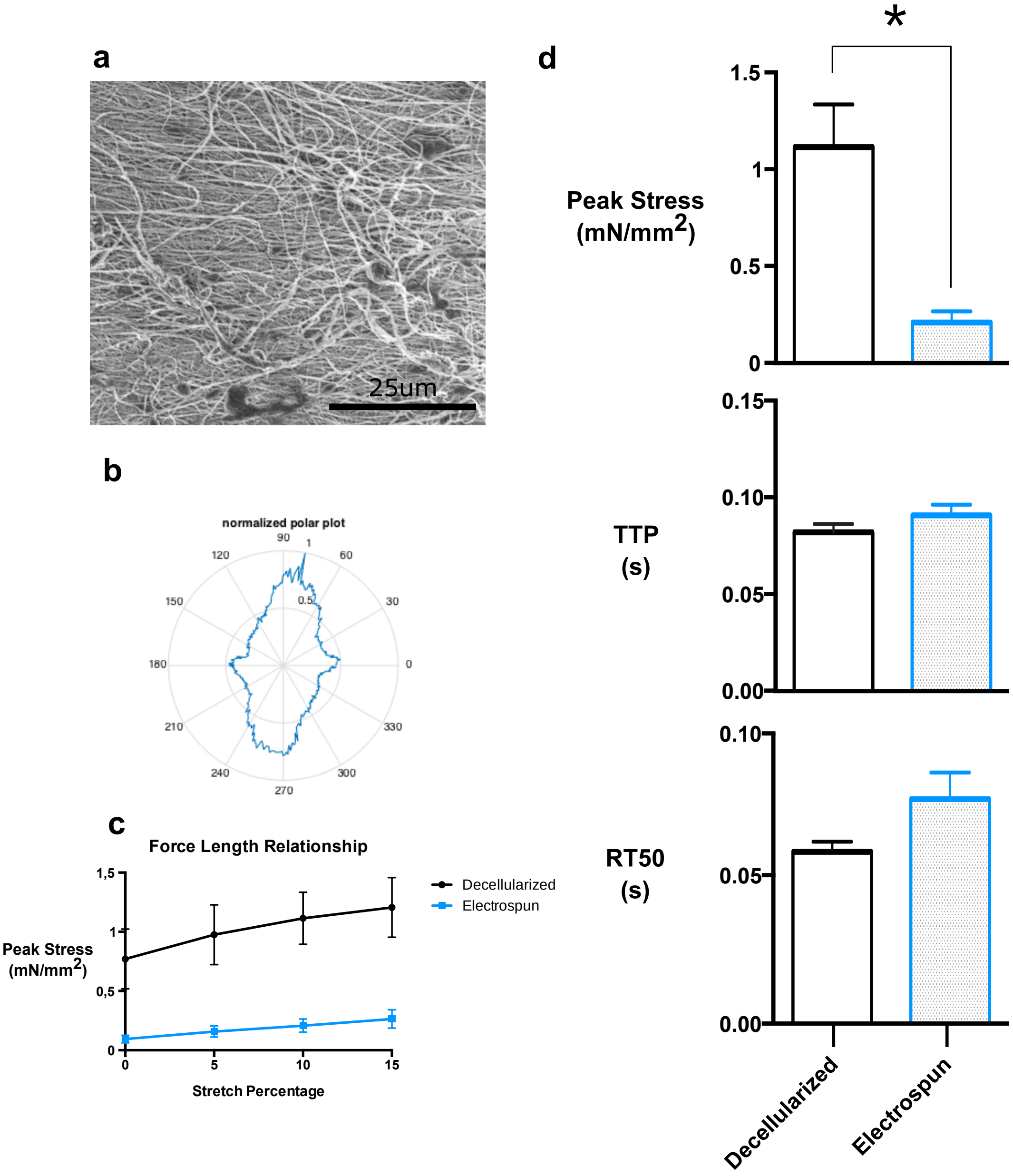

Supplementary Figure 4 | Scaffold comparison study using NRVM cells
Anisotropic electrospun gelatin scaffolds were compared with laser-cut decellularized myocardium. **(a)** SEM image showing the anisotropic fibers of the electrospun scaffold, Scale bar is 25 µm. **(b)** Using Fast-Fourier- Transform and Matlab image processing fiber alignment was determined. **(c)** At day 9 post-seeding, laser-cut decellularized myocardium (black, n=6) and anisotropic electrospun gelatin scaffolds (blue, n=7) exhibited a positive Frank- Starling response. **(d)** Measurements of peak stress, time to peak (TTP) and relaxation time (RT50) in both scaffold types (Paired Student's t-test, *$p<0.01$)

a

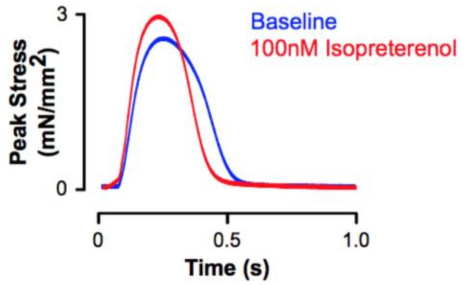

b

	Baseline	100nM Isoproterenol
Peak Stress (mN/mm^2)	2,2 ± 0.76	2,5 ± 0.83
RT 50 (ms)	168 ± 10	122 ± 11 **
TTP (ms)	181 ± 12	155 ± 11*

Supplementary Figure 5 │ β-adrenergic response of hiPSC-CM EHTs

Lusitropic behavior of hiPSC-CM EHTs upon β-adrenergic agonist treatment (**a**) Representative traces of one 16 day old EHT at baseline and after treatment with 100nM Isoproterenol. (**b**) Mean twitch kinetic parameters and peak tension measured in hiPSC-EHTs (n = 8) at baseline and after treatment with 100nM of Isoproterenol (Paired Student's t-test, *$p<0.05$, **$p<0.001$)

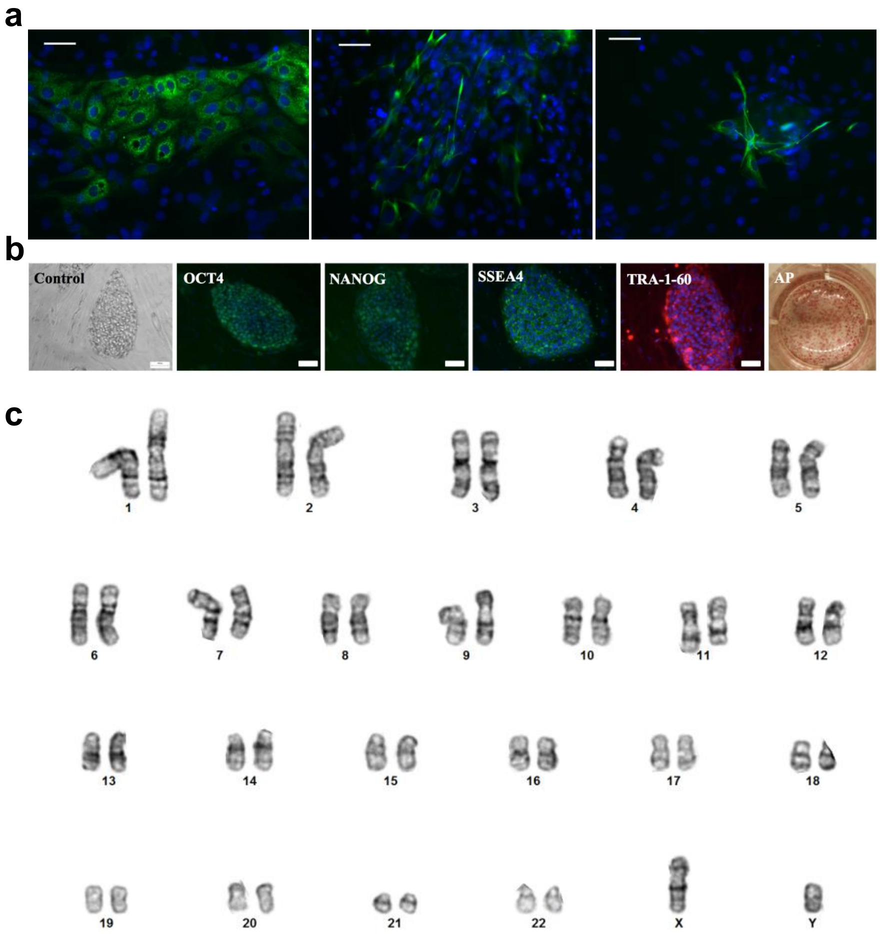

Supplementary Figure 6 | Evaluation of pluripotency of iPSC
(**a**) Stains for AFP (Endoderm), Desmin (Mesoderm), Nestin (Ectoderm) scale bar is 50μm. (**b**) hiPSC line derived from a healthy control subject showing pluripotency markers. (**c**) Normal karotype for the derived hiPSC line.

Supplementary Table 1 │Detailed fold change values of BNP up or down-regulation upon applied stretch or shear (in comparison to control). Expressions were normalized to GAPDH. Each batch of seeded tissues was analyzed within itself.

Control		Stretch		Shear	
NRVM	iPSC-CM	NRVM	iPSC-CM	NRVM	iPSC-CM
1,407	0,994	1,324	1,241	1,290	0,718
0,711	1,006	1,782	0,911	0,745	0,518
1,949	0,781	1,156	3,963	1,040	0,597
0,660	1,274	0,960	1,485	1,000	0,654
0,777	0,653	1,230	2,181	1,233	
1,563	1,540	0,784	5,756	0,591	
1,162	0,652	2,168	3,543	0,779	
0,551	0,302	1,221	2,819	0,839	
2,240		1,937	0,524	1,385	
0,920		2,421		1,476	
0,485		2,042		0,813	
1,475		0,790		0,432	
1,454		1,170		1,993	
1,016		1,071		1,709	
0,544		1,191		0,484	
0,842		0,945		0,501	
		1,070		0,689	
		1,446		0,665	
				0,620	
				0,580	
				0,547	
				1,292	

Bibliography

1. Gui, L., Chan, S. A., Breuer, C. K. & Niklason, L. E. Novel utilization of serum in tissue decellularization. *Tissue Engineering Part C: Methods* **16,** 173–184 (2010).
2. Schroder, E., Byse, M. & Satin, J. L-Type Calcium Channel C Terminus Autoregulates Transcription. *Circ Res* **104,** 1373–1381 (2009).
3. Bartulos, O. *et al.* ISL1 cardiovascular progenitor cells for cardiac repair after myocardial infarction. *JCI Insight* **1,** 1–17 (2016).
4. Lian, X. *et al.* Directed cardiomyocyte differentiation from human pluripotent stem cells by modulating Wnt/β-catenin signaling under fully defined conditions. *Nature Protocols* **8,** 162–175 (2013).
5. Korff, T. & Augustin, H. G. Integration of endothelial cells in multicellular spheroids prevents apoptosis and induces differentiation. *The Journal of Cell Biology* **143,** 1341–1352 (1998).
6. Kobsa, S. *et al.* An electrospun scaffold integrating nucleic acid delivery for treatment of full-thickness wounds. *Biomaterials* **34,** 3891–3901 (2013).
7. Song, J.-H., Kim, H.-E. & Kim, H.-W. Production of electrospun gelatin nanofiber by water-based co-solvent approach. *J Mater Sci Mater Med* **19,** 95–102 (2008).
8. Sisson, K., Zhang, C., Farach-Carson, M. C., Chase, D. B. & Rabolt, J. F. Evaluation of cross-linking methods for electrospun gelatin on cell growth and viability. *Biomacromolecules* **10,** 1675–1680 (2009).
9. Chomczynski, P. & Mackey, K. Substitution of chloroform by bromo-chloropropane in the single-step method of RNA isolation. *Analytical Biochemistry* **225,** 163–164 (1995).
10. Livak, K. J. & Schmittgen, T. D. Analysis of relative gene expression data using real-time quantitative PCR and the 2(-Delta Delta C(T)) Method. *Methods* **25,** 402–408 (2001).
